# Total neoadjuvant therapy in locally advanced rectal cancer: Role of systemic chemotherapy

**DOI:** 10.1002/ags3.12253

**Published:** 2019-04-29

**Authors:** Ri Na Yoo, Hyung Jin Kim

**Affiliations:** ^1^ Division of Colorectal Surgery Department of Surgery St. Vincent's Hospital College of Medicine The Catholic University of Korea Suwon Gyeonggi‐do Korea

**Keywords:** chemoradiation, chemotherapy, neoadjuvant therapy, organ preservation, rectal cancer

## Abstract

For the past several decades, disease‐related outcomes, particularly local recurrence rate, in patients with locally advanced rectal cancer have significantly improved as a result of advancement of surgical technique and implementation of neoadjuvant chemoradiation. However, distant metastasis remains unresolved, being a significant cause of cancer death. To focus on micrometastases early in the course of multimodal treatment, delivering systemic chemotherapy in the neoadjuvant setting is emerging. Also, driven by patient demand and interest in preserving quality of life, upfront chemotherapy prior to surgery serves as a strategy for organ preservation in the management of rectal cancer. Herein, currently available literature on different methods and strategies of the multimodal approach is critically appraised.

## 1 INTRODUCTION

The multidisciplinary approach of neoadjuvant chemoradiotherapy (nCRT) followed by total mesorectal excision (TME) and postoperative adjuvant chemotherapy has been accepted worldwide as the standard treatment for patients with locally advanced rectal cancer. This therapeutic approach markedly reduced the local recurrence rate from 35% to 5%‐10% and significantly improved overall survival.^1^, ^2^, ^3^, ^4^ Local recurrence now seems less of a concern, whereas distant metastasis is now the leading cause of cancer death in rectal cancer.^5^ Although adjuvant chemotherapy has been recommended in patients with locally advanced rectal cancer treated with nCRT and TME, it does not show clear benefit in improving overall survival or cancer‐specific survival.^6^ Poor patient compliance with adjuvant chemotherapy is also a serious concern in clinical practice.^6^, ^7^ Therefore, the efficacy of adjuvant chemotherapy in rectal cancer treatment remains controversial.

With the purpose of improving patient survival, delivery of chemotherapy before surgery had been proposed to treat occult micrometastases early and increase treatment compliance.^8^ Multiple trials evaluating various modes of incorporating both chemotherapy and CRT in the neoadjuvant setting, referred to as “total neoadjuvant therapy (TNT),” have reported optimistic results. This review discusses the rationale for TNT and available evidence from clinical trials, appraising the emerging literature on the conception of organ preservation to the current therapeutic paradigm.

## 2 CHRONOLOGY OF THE MULTIMODAL APPROACH IN RECTAL CANCER TREATMENT

In the 1980s, reports by Heald et al, including a report on the “Holy Plane” by Heald alone, were acclaimed in the history of rectal cancer surgery.^9^, ^10^ Dissecting the entire rectum through the “Holy Plane,” meaning the surgical plane based on the embryological development of the hindgut, resulted in removal of the entire circumferential perirectal tissue without leaving mesorectal tissue.^10^ This artful surgical technique reduced the rate of positive circumferential resection margin; consequently, the local recurrence rate dropped dramatically from 30%‐50% to a single digit, and the survival outcome ultimately improved up to 80%.^1^ TME also allowed patients to undergo a sphincter‐saving procedure.^11^ Now, the prime role of TME in rectal cancer management is indisputable.

**Figure 1:**
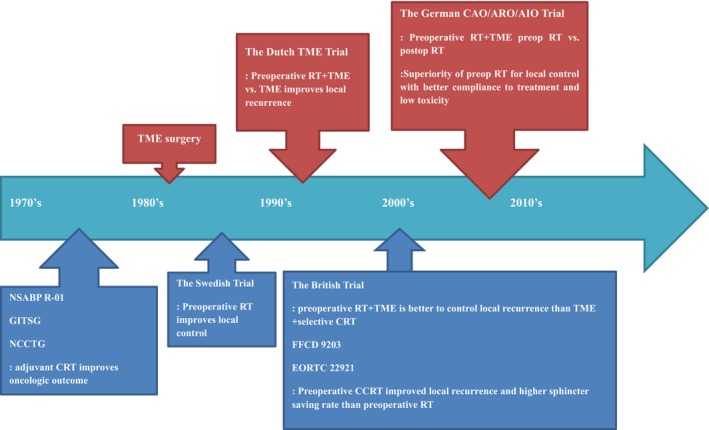
Chronology of major research results in rectal cancer management. CRT, chemoradiotherapy; EORTC, European Organisation for Research and Treatment of Cancer Radiotherapy Group; FFCD, The Fédération Francophone de Cancérologie Digestive; GITSG, Gastrointestinal Tumor Study Group; NCCTG, North Central Cancer Treatment Group; NSABP, National Surgical Adjuvant Breast and Bowel Project; RT, Radiation therapy; TME, total mesorectal excision

At a similar time, the effectiveness of chemotherapy and radiation therapy (RT) was eagerly investigated for the treatment of locally advanced rectal cancer as shown in Figure 1. Before TME had gained popularity and general application in rectal cancer surgery, early clinical trials, such as NSABP R‐01, NCCTG or GITSG G‐7175, were conducted to test whether postoperative RT, chemotherapy, or combined chemoradiotherapy could improve survival benefit with effective local control.^12^, ^13^, ^14^ These trials indicated that adjuvant radiotherapy could improve local control and, in a combined setting with chemotherapy, possible improvement in survival was suggested. Based on these findings, adjuvant chemotherapy with radiotherapy was recommended in the management of rectal cancer.^15^

Although the trials of adjuvant therapy in a postoperative setting were mainly conducted in the USA, preoperative radiation therapy was common in European countries. The Swedish Trial evaluating preoperative short‐course RT combined with conventional rectal resection demonstrated that both the 5‐year local recurrence rate and the 5‐year survival rate significantly improved with preoperative short‐course RT, 9% vs 23% and 58% vs 48% respectively, among the curatively treated patients.^16^ Along with implementation of TME, the Dutch TME trial showed a significant benefit of preoperative RT in reducing the local recurrence rate for patients with TNM stage III.^5^ However, the overall survival or cancer‐specific survival was similar when compared with the patients who received TME surgery only.

In terms of sequencing multimodal therapy, the German trial clearly showed the superiority of preoperative RT over postoperative therapy.^17^ Similar trials indicated that preoperative radiotherapy combined with TME effectively lowered the risk of pelvic local recurrence, while high patient compliance with and low toxicity of the treatment were obtained.^18^, ^19^, ^20^ Subsequent trials showed that concurrent chemotherapy with fluorouracil and leucovorin, acting as a radiosensitizer, significantly boosted the effect of preoperative radiotherapy.^2^, ^3^, ^17^, ^21^ Consequently, the rate of local recurrence reached as low as 5.3%, and sphincter‐saving surgery was possible in the majority of patients. Thus, the standard of care in most countries has been preoperative chemoradiotherapy followed by TME surgery. Nonetheless, the 10‐year cumulative incidence of distant metastases was 30% and the 10‐year disease‐free survival was 68% overall.^17^ It is clear that better systemic control is necessary.

## 3 LIMITATION OF POSTOPERATIVE ADJUVANT CHEMOTHERAPY

Adjuvant chemotherapy has been a standard part of the multimodal approach in the management of locally advanced rectal cancer and has been recommended in the US National Comprehensive Cancer Network (NCCN) guidelines. This recommendation for patients with stage II or III rectal cancer is based on extrapolation of results from phase III trials of adjuvant treatment for colon cancer^22^, ^23^ and from data of patients with rectal cancer treated without preoperative radiotherapy or CRT.^24^ According to a systematic review and meta‐analysis of adjuvant chemotherapy after preoperative radiotherapy and surgery in patients with rectal cancer, adjuvant chemotherapy for rectal cancer does not improve overall survival, disease‐free survival, or distant recurrences.^6^, ^25^ However, in a subgroup analysis of patients with tumors 10‐15 cm from the anal verge, there was improved disease‐free survival and fewer distant metastases with adjuvant therapy.^6^ Another meta‐analysis suggested that patients with a pathological complete response (pCR) after chemoradiotherapy might not benefit from adjuvant chemotherapy, whereas patients with residual tumor showed superior outcomes when adjuvant chemotherapy was given.^26^ Such conflicting interpretation of available data in the benefit of adjuvant chemotherapy has resulted in endless debates on its clinical application.

In addition, serious concerns regarding patient compliance with adjuvant therapy was raised. Nearly 30% of eligible patients had never initiated adjuvant chemotherapy^27^ and less than half of them had received the full treatment without interruption or delays.^7^, ^18^, ^28^ Postoperative complications including leakage, poor general condition and slow recovery, problems with temporary stoma, or refusal of treatment were the main reasons for withdrawal from adjuvant therapy.^29^ Evaluation on the timing and efficacy of postoperative adjuvant chemotherapy showed that each 4‐week delay in treatment correlated with a 14% drop in overall survival.^30^ It is clear that poor treatment compliance with adjuvant chemotherapy, regardless of the reasons, impedes patient survival. Therefore, other modes of delivering chemotherapy that can increase compliance are desperately needed. Hence, the concept of delivering chemotherapy before surgery is proposed to treat occult micrometastases early and increase treatment compliance, ultimately improving survival outcome.^8^ Different methods and schedules to carry out systemic chemotherapy before surgery are now of particular interest in clinical trials of rectal cancer management.

## 4 CLINICAL IMPORTANCE OF PATHOLOGICAL COMPLETE RESPONSE AFTER nCRT

It is well known that nCRT not only results in significant downsizing or downstaging of tumor, but also in pathological complete response (pCR) defined as the complete absence of cancer cells in the resected specimen. In trials evaluating nCRT in locally advanced rectal cancer, pCR was reported in 10%‐32% of patients.^31^, ^32^, ^33^ In general, patients who were treated with nCRT and TME did not show significant improvement in overall survival despite the excellent local control rate.^12^, ^34^ However, the prognosis in patients who obtain pCR is usually excellent, so that it is often used as a surrogate marker for good oncological outcome.^35^

Analysis of the factors that might increase the rate of pCR showed that the use of continuous infusion of 5‐fluorouracil, the addition of a second cytotoxic drug and high radiation dose were associated with a higher rate of pCR.^31^, ^32^ Noninferiority, phase III randomized trials comparing capecitabine to 5‐fluorouracil in CRT showed that capecitabine could be an alternative in neoadjuvant or adjuvant CRT regimens.^36^ Given that capecitabine functions equally as a radiosensitizer, addition of oxaliplatin to either continuous 5‐fluorouracil or capecitabine and radiotherapy during chemoradiation was attempted.^37^, ^38^, ^39^ Unfortunately, these trials failed to show improvement of tumor response but only increased toxicity. In terms of the long‐term oncological outcomes, conflicting results were reported. In the ACCORD 12 trial, the addition of oxaliplatin did not show significant improvement in 5‐year disease free survival or overall survival.^40^ However, in the German CAO/ARO/AIO‐04 trial, 3‐year disease‐free survival was significantly enhanced.^41^ Nevertheless, at this time, the addition of oxaliplatin to standard nCRT is not supported. Finally, although the rate of pCR was higher with increasing radiation dose, a dose‐response effect beyond 45 Gy was not recommended due to a lack of data.^31^, ^32^ Therefore, 5‐fluoropyrimidine‐based nCRT remains the current standard treatment, and a way of intensifying the effect of nCRT is the primary focus of ongoing clinical trials.

One problem related to the identification of patients who have achieved pCR is the optimal interval from the end of chemoradiation to surgery. It is known that the effect of chemoradiation takes time for a result to be seen, and complete clinical regression of tumor could be achieved when surgery occurred after a longer time interval.^42^ The Lyon R90‐01 trial showed that patients undergoing surgery at 6‐8 weeks after completion of nCRT had significantly increased rates of pCR compared to those operated at 2 weeks after the completion of nCRT, 26% vs 10% respectively, supporting the currently accepted timing of surgery.^43^ The 17‐year follow‐up report indicated that the long‐term oncological outcome was similar in the two groups: local recurrence was 14% in the 6‐ to 8‐week group and 12% in the 2‐week group, and the overall survival was 42% and 40%, respectively.^33^ With a longer interval after therapy, tumor shrinkage and downstaging seemed to be more apparent. Other retrospective studies and meta‐analyses also support that tumor response to nCRT is time‐dependent and suggest that an extended interval between completion of nCRT and surgery is an important determinant in achieving a pCR.^44^, ^45^, ^46^, ^47^ However, the GRECCAR‐6 trial, a phase III randomized, multicenter trial evaluating the effect of interval (7 or 11 weeks) between nCRT and surgery on pCR, failed to show a significant difference in the pCR rate between the two groups.^48^ The authors concluded that the longer interval resulted in increased postoperative morbidity and poor quality of mesorectal resection without increasing pCR rate. Furthermore, the possibility of preventing disease progression and distant metastasis by many clinicians and surgeons is not yet fully addressed. The optimal interval of the waiting period and the timing of surgery remain to be clarified. Further research is needed to determine the risk and benefit of a prolonged interval between nCRT and surgery. Nevertheless, to improve pCR rates, upfront chemotherapy prior to surgery is under active investigation. The question of timing of surgery may receive clues from the period of upfront chemotherapy.

## 5 MODE OF DELIVERING SYSTEMIC CHEMOTHERAPY: CONSOLIDATION CHEMOTHERAPY

In order to address the risk of disease progression and distant metastasis during a longer interval between nCRT and surgery and to improve tumor regression, delivering systemic chemotherapy after nCRT, called consolidation chemotherapy, was suggested. Currently available reports on consolidation chemotherapy are listed in Table 1.^49^, ^50^, ^51^, ^52^ A prospective, multicenter, phase II trial was conducted to evaluate the effect of adding cycles of chemotherapy in between chemoradiation and surgery, extending the waiting period.^49^ This study showed that the pCR rate increased with the length of chemoradiation‐to‐surgery interval and with increased number of chemotherapy cycles. Surgical complications or surgical technical difficulty after delaying surgery for up to 20 weeks was not increased, showing surgical safety. Despite long‐term oncological outcome not being reported, disease progression or distant metastasis was not observed, which implies that consolidation chemotherapy is oncologically safe. Treatment compliance with neoadjuvant chemotherapy was only as low as 77%, which is much better compared to 40%‐60% for the adjuvant chemotherapy.^6^, ^7^ What is uncertain in this trial is the relative contribution of chemotherapy and waiting period to the rate of pCR. This ambiguity is expected to be clarified when the results from the RAPIDO phase III randomized trial, comparing short‐course radiotherapy followed by capecitabine and oxaliplatin chemotherapy before surgery to standard long‐course nCRT, are received.^53^

**Table 1:** Studies investigating consolidation chemotherapy

Study	Design	N	CRT regimen	NAC regimen	Adjuvant CTx	pCR rate (%)	Compliance	R0 resection rate (%)	Surgical complication rate (%)	Survival outcome
Garcia‐Aguilar^49^	Phase II nonrandomized four‐arm	259	CRT+5FU	None	mFOLFOX (8×)	18	Not reported	98	15	Not reported
			CRT+5FU	mFOLFOX6 (2×)	mFOLFOX (6×)	25	82% completed NAC	100	6	Not reported
			CRT+5FU	mFOLFOX6 (4×)	mFOLFOX (4×)	30	81% completed NAC	96	4	Not reported
			CRT+5FU	mFOLFOX6 (6×)	mFOLFOX (2×)	36	77% completed NAC	100	9	Not reported
Polish II trial^50^	Phase III randomized two‐arm	515	RT (5 × 5 Gy)	FOLFOX4 (3 cycles)	Not reported	16	63% completed NAC	77	29	3‐y DFS = 53% 3‐y OS = 73%
			CRT+5FU / leucovorin / oxaliplatin	None	Not reported	12	66% completed CRT	71	25	3‐y DFS = 52% 3‐y OS = 65%
Gao^51^	Prospective single‐arm	36	CRT+CAPOX	CAPOX (1×)	Not reported	36	94% completed NAC	100	36	Not reported
Zhu^52^	Phase II single‐arm	42	CRT+CAPOX	Cape (1×)	CAPOX (6‐8×)	17	100% completed NAC	92	16	3‐y DFS = 57% 3‐y OS = 66%

5‐FU, 5‐fluorouracil; Cape, capecitabine; CAPOX, capecitabine/oxaliplatin; CRT, chemoradiotherapy; CTx, chemotherapy; DFS, disease‐free survival; Gy, gray; mFOLFOX6, 5‐fluorouracil, leucovorin, and oxaliplatin; NAC, neoadjuvant chemotherapy; OS, overall survival; pCR, pathological complete response; R0, microscopically clear resection; RT, radiotherapy.

A large phase III Polish trial provides valuable information regarding the long‐term oncological outcome after consolidation chemotherapy.^50^ In this trial, patients were randomly assigned to two treatment groups: one with short‐course radiotherapy followed by three cycles of chemotherapy with fluorouracil and oxaliplatin and the other with the standard CRT with oxaliplatin and boluses of 5‐fluorouracil and leucovorin. Both groups had similar intervals between the start of irradiation and surgery, approximately 12 weeks. The R0 resection rate and postoperative surgical complication rate were similar in both groups. Also, there was no difference in disease‐free survival, local failure rate, and distant metastases rate in the two groups. However, the patients given short‐course radiotherapy followed by consolidation chemotherapy showed better treatment compliance and lower acute toxicity than the long‐course CRT group. Interestingly, the 3‐year overall survival was better in the patients with short‐course radiotherapy followed by consolidation chemotherapy, despite the rate of distant metastasis or local recurrence being similar in the two groups. The authors presumed that large irradiation doses result in activation of antitumor immune responses during the waiting period before surgery. Observation on longer follow ups will probably clarify this phenomenon. So far, these studies suggest that consolidation chemotherapy can be a safe and feasible option in the multimodal approach, encouraging implementation in future trials.

## 6 MODE OF DELIVERING SYSTEMIC CHEMOTHERAPY: INDUCTION CHEMOTHERAPY

Another way of delivering chemotherapy before surgery is dividing adjuvant chemotherapy and delivering a limited number of cycles before nCRT, and then delivering the remaining cycles postoperatively, named induction therapy.^54^ Studies assessing the effect of induction chemotherapy are listed in Table 2.^55^, ^56^, ^57^, ^58^, ^59^, ^60^, ^61^, ^62^ This approach is particularly appealing for locally far advanced diseases showing high‐risk features of distant metastasis or difficult local resection, such as extramural venous invasion or lateral pelvic lymph node metastasis. Theoretically, upfront chemotherapy allows the chemotherapeutic agents to reach the primary tumor directly when the vasculature is not disrupted by either radiation or surgery. Therefore, the tumor may optimally respond to the chemotherapeutic agent. Clinical trials of induction chemotherapy show no adverse effect that would delay treatment, increased pCR rate, and early identification of nonresponders along with excellent treatment compliance.^8^, ^31^, ^35^, ^54^ Regarding long‐term oncological outcome, the Spanish GCR‐3 phase II trial reported that local recurrence rate and survival outcomes at 5 years of follow up were not different from the control group, although the experimental group showed lower toxicity and better compliance.^55^ The CONTRE trial notably demonstrated a similar pCR rate to the consolidation chemotherapy trial conducted by Garcia‐Aguilar et al.^57^ This prospective single‐arm study reported the outcome of patients who received eight cycles of mFOLFOX6 before CRT. Again, the compliance rate was over 90%, and the R0 resection rate was 100%. It appears that approximately 6‐8 cycles of induction chemotherapy are needed to achieve a similar rate of pCR. In a future trial, the optimal duration and number of cycles of induction chemotherapy would be an important topic to address.

**Table 2:** Studies investigating induction chemotherapy

Study	Design	N	NAC regimen	CRT regimen	Adjuvant CTx	pCR rate (%)	Compliance	R0 resection rate (%)	Survival outcome
Spanish GCR‐3^55^	Phase II RCT	108	CAPOX (4×)	CRT+CAPOX		14	94% completed NAC; 85% completed CRT	86	5‐y DFS = 64% 5‐y OS = 74%
					CAPOX (4×)	13	57% completed adjuvant CTx; 80% completed RT	87	5‐y DFS = 62% 5‐y OS = 77%
EXPERT^56^	Phase II single‐arm	105	CAPOX (4×)	CRT+Cape	Cape (12 wks)	20	89% complete NAC; 91% complete CRT	98	5‐y DFS = 64% 5‐y OS = 75%
CONTRE^57^	Prospective single‐arm	39	mFOLFOX6 (8×)	CRT+5FU or Cape	None	33	92% completed NAC; 90% completed CRT	100	Not reported
COPERNICUS^58^	Phase II single‐arm	60	Oxali+5‐FU (4×)	SC‐RT (5 Gy × 5)	Oxali+5‐FU (8×)	12	95% completed NAC; 97% completed CRT	98	2‐y PFS = 86.2%
Dueland^59^	Phase II single‐arm	97	FLOX (2×)	CRT+CAPOX	None	17	98% completed NAC; 95% completed RT	90	5‐y DFS = 61% 5‐y OS = 83%
Schou^60^	Prospective single‐arm	84	CAPOX (2×)	CRT+Cape	Not reported	23	91% completed 21 NAC cycles; 93% completed CRT	94	5‐y DFS = 63% 5‐y OS = 67%
EXPERT‐C^61^	Phase II RCT	165	CAPOX+cetuximab (4×)	CRT+Cape+ cetuximab	CAPOX+cetuximab (4×)	11	95% completed NAC; 91% completed CRT	96	Not reported
			CAPOX (4×)	CRT+Cape	CAPOX (4×)	7	93% completed NAC; 90% completed CRT	92	Not reported
AVACROSS^62^	Phase II single‐arm	47	CAPOX1 Bevacizumab (4×)	CRT+Cape+ bevacizumab	CAPOX (4×)	34	85% completed NAC; 83% completed CRT	98	Not reported

5‐FU, 5‐fluorouracil; Cape, capecitabine; CAPOX, capecitabine/oxaliplatin; CRT, chemoradiotherapy; CTx, chemotherapy; DFS, disease‐free survival; Gy, gray; mFOLFOX6, 5‐fluorouracil, leucovorin, and oxaliplatin; NAC, neoadjuvant chemotherapy; OS, overall survival; pCR, pathological complete response; PFS, progression‐free survival; R0, microscopically clear resection; RT, radiotherapy; SC‐RT, short‐course radiotherapy.

Two clinical studies included target agents in the induction therapy. EXPERT‐C, a branching trial of EXPERT, investigated the effect of adding cetuximab to capecitabine‐based induction chemotherapy followed by chemoradiotherapy, then again adding cetuximab in adjuvant chemotherapy.^61^ Similar to other studies, this randomized trial indicated that compliance was over 90% in both groups, but either pCR rate or R0 resection in the two groups was not different. AVACROSS was a phase II single‐arm study evaluating the effect of bevacizumab to induction chemotherapy with capecitabine and oxaliplatin followed by chemoradiotherapy.^62^ Along with high compliance with the treatment, the pCR rate was as high as 34%, and the R0 resection rate was 98%. However, postoperative morbidity occurred in 58% of patients, and 24% required surgical reintervention. The safety of adding bevacizumab to induction chemotherapy should be addressed. Nevertheless, the role of target agent in induction chemotherapy is not yet fully investigated.

## 7 RISK‐ADAPTED APPROACH AND ORGAN PRESERVATION

Based on previous reports, it is quite certain that TNT, consolidation or induction chemotherapy with nCRT, provides several benefits in the treatment of locally advanced rectal cancer. It not only improves pCR rate and treatment compliance but also delivers early systemic control for possible micrometastasis. Beyond these benefits, TNT gives the opportunity to assess chemosensitivity and tumor response prior to surgery. This can lead to risk stratification and identification of patients who may not require surgery or radiation therapy. In fact, several studies reporting a nonoperative approach have suggested that with robust follow ups, a certain subgroup of patients who achieved a complete clinical response could be safely left with the rectum and have good long‐term oncological outcomes.^63^, ^64^, ^65^ However, an important concern arising from this “watch and wait” approach is how to accurately access tumor regression and identify patients with no residual disease.

**Figure 2:**
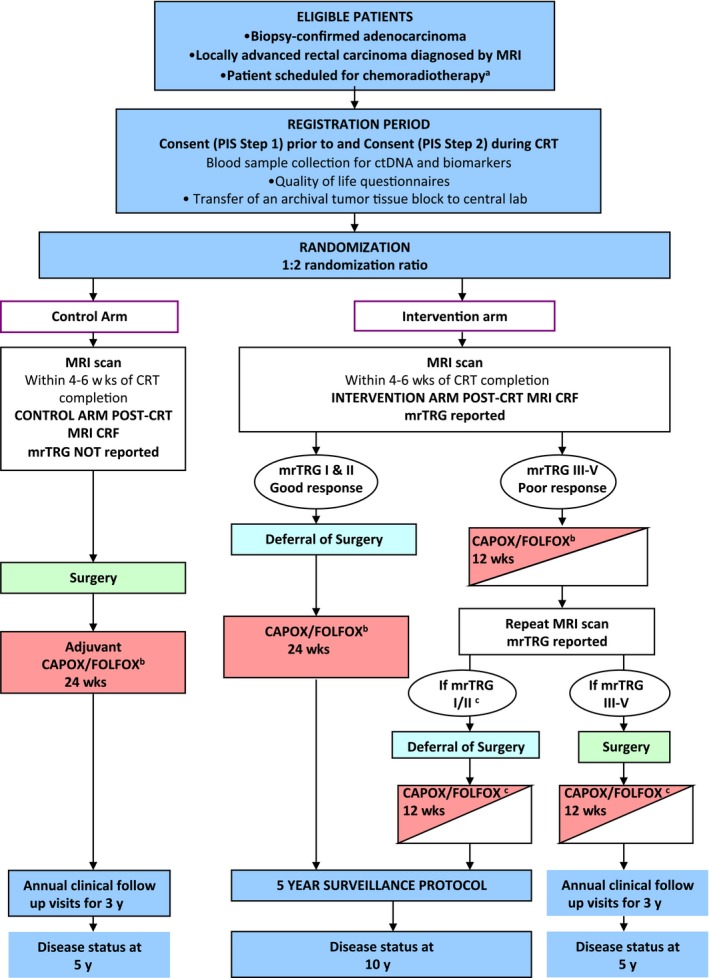
The TRIGGER trial study schema. The TRIGGER trial is a multicenter, open, interventional, randomized control feasibility study to validate assessment of tumor response based on a magnetic resonance imaging (MRI)‐derived tumor regression grading system, named mrTRG.^67^ CRF, clinical report form; CRT, chemoradiotherapy; PIS, patient information sheet

Reflecting back to the past, pelvic magnetic resonance imaging (MRI) enormously contributed to the development of a multidisciplinary therapeutic approach, providing accurate clinical staging. Pelvic MRI has been used as a tool to guide preoperative decision on treatment modality, essentially determining which patients undergo nCRT before surgery.^66^, ^67^ Also, previous studies show that high‐resolution MRI can predict the survival outcome based on the assessment of tumor regression grade and circumferential resection margin status prior to surgery, enabling identification of poor responders and good responders.^68^, ^69^ Thus, MRI can help to appropriately stratify risk and identify potential candidates for organ preservation. The TRIGGER trial, a multicenter, open, interventional, randomized control feasibility study, is ongoing to validate assessment of tumor response based on an MRI‐derived tumor regression grading system named mrTRG for short.^70^ Shown in Figure 2, two prospective subtrials comprise the intervention arm based on mrTRG: the good‐response group and the poor response group. The good response group follows a nonoperative approach with additional systemic chemotherapy, whereas the poor response group is planned for upfront systemic chemotherapy before surgery. Results from this study will provide much vital information regarding the role of MRI in risk‐adapted approach.

A large phase II multicentered randomized trial is also ongoing investigating the efficacy of TNT and a selective nonoperative approach in locally advanced rectal cancer.^71^ In this trial, patients are randomly assigned to either an induction chemotherapy or a consolidation chemotherapy group. Upon completion of neoadjuvant therapy, restaging with digital rectal exam, endoscopy, and MRI will be undertaken to measure tumor regression and apply risk stratification to determine whether patients should undergo standard TME surgery or nonoperative management. This study will provide information on 3‐year recurrence‐free survival in patients undergoing selective nonoperative management.

Apart from nonoperative management, there is an attempt to omit radiotherapy in the course of TNT. Several small, single‐arm studies reported that neoadjuvant chemotherapy alone could result in relatively comparable oncological outcome and, in some patients, even tumor downstaging and complete regression occurred.^72^, ^73^, ^74^ A phase II pilot study of induction chemotherapy without radiotherapy in locally advanced rectal cancer showed that R0 resection was fully achieved in all patients, and the pCR rate to chemotherapy alone was 25%.^75^ Furthermore, the oncological outcome was not compromised: 0% for the 4‐year local recurrence rate and 84% for the 4‐year disease‐free survival rate. A multicentered, phase III randomized controlled trial, Preoperative Radiation or Selective Preoperative Radiation and Evaluation Before Chemotherapy and TME (PROSPECT) is under investigation to validate the findings of the pilot study.

Several clinical trials, listed in Table 3, are ongoing, investigating the effectiveness and efficacy of the TNT approach.^53^, ^70^, ^71^, ^75^, ^76^, ^77^, ^78^ All these trials uniformly show the immense effort that is being made to intensify neoadjuvant therapy and to improve survival outcome. Outcomes of the trials will help understand tumor characteristics and provide essential information on optimization of the TNT approach. The results of these studies are earnestly anticipated.

**Table 3:** Ongoing trials investigating the TNT approach and organ preservation

TNT type	Trial	Design	N	Arms	1° endpoint	2° endpoint
Consolidation	RAPIDO trial^53^ (NCT01558921)	Phase III RCT	842	Standard long course CRT → surgery → optional adjuvant CAPOX (8×)	3‐y DFS	Toxicity, R0 resection rate, pCR QOL, functional outcome OS
				SC‐RT (5 Gy × 5) → CAPOX (6×) → surgery		
Consolidation	TRIGGER trial^70^ (NCT02704520)	Phase III RCT	633	Refer to Figure 2	Rate of patient recruitment and randomization	Rate of unit recruitment, toxicity, reproducibility of mrTRG reporting, surgical morbidity, pCR, residual tumor density, surgical quality rates
Consolidation or induction	Smith et al^71^ (NCT02008656)	Phase II RCT	202	Induction CTx+CRT	3‐y RFS	Organ preservation rate, compliance, toxicity, functional outcome, QOL
				CRT+consolidation CTx		
Consolidation	KONCLUDE^76^ (NCT02843191)	Phase III RCT	358	Standard CRT→ surgery → mFOLFOX6 (8×)	pCR 3‐y DFS	Toxicity, R0 resection rate, tumor response rate, postoperative morbidity, peripheral neuropathy at 3 y after surgery
				Standard CRT → mFOLFOX6 (3×) → surgery → mFOLFOX (5×)		
TNT without RT	PROSPECT^75^ (NCT01515787)	Phase III RCT	1060	5‐FU +CRT → surgery → FOLFOX (8×)	R0 rate DFS LRR	pCR OS Toxicity Rate of CRT
				FOLFOX (6×) → tumor response assessment → TME or CRT Adjuvant therapy if R0 → FOLFOX (6×) R1+ → FOLFOX (4×)+CRT		
TNT without RT	BACCHUS^77^ (NCT01650428)	Phase II RCT	60	FOLFOX + bev	pCR rate	Response rate CRM negative resection T and N downstaging PFS, DFS, OS, LRR 1‐y colostomy rate Toxicity, compliance
				FOLFOXIRI + bev		
TNT with or without RT	FOWARC^78^ (NCT01211210)	Phase II RCT	495	Standard CRT	3‐y DFS	pCR, R0, LRR, OS Predictive biomarkers QOL, toxicity
				FOLFOX + CRT		
				FOLFOX alone		

5‐FU, 5‐fluorouracil; bev, bevacizumab; Cape, capecitabine; CAPOX, capecitabine/oxaliplatin; CRM, circumferential resection margin; CRT, chemoradiotherapy; CTx, chemotherapy; DFS, disease‐free survival; FOLFOXIRI, oxaliplatin/5‐FU/irinotecan; Gy, gray; LRR, local recurrence rate; mFOLFOX6, 5‐fluorouracil, leucovorin, and oxaliplatin; mrTRG, magnetic resonance imaging‐derived tumor regression grading system; OS, overall survival; pCR, pathological complete response; PFS, progression‐free survival; QOL, quality of life; R0, microscopically clear resection; RCT, randomized controlled trial; RT, radiotherapy; SC‐RT, short‐course radiotherapy; TME, total mesorectal excision; TNT, total neoadjuvant therapy.

## 8 CONCLUSION

Total neoadjuvant therapy offers a chance to deliver aggressive treatment against the development and progression of micrometastases, potentially increasing survival rates in locally advanced rectal cancer. Furthermore, there is tremendous interest and desire for organ preservation in rectal cancer partly driven by patients who want to preserve a decent quality of life in the modern era. The total neoadjuvant therapy approach may facilitate a greater number of patients having the potential for organ preservation. Upcoming results from multiple ongoing and future trials will assist the clinical decisions that fulfil optimal oncological outcomes as well as quality of life.

## DISCLOSURE

Conflicts of Interest: Authors declare no conflicts of interest for this article.

Author Contribution: Hyung Jin Kim and Ri Na Yoo conceived of the presented idea. Hyung Jin Kim developed the theory. Ri Na Yoo drafed the manuscript. Hyung Jin Kim and Ri Na Yoo both discussed the final draft of manuscript.
